# Primaquine Inhibits the Endosomal Trafficking and Nuclear Localization of EGFR and Induces the Apoptosis of Breast Cancer Cells by Nuclear EGFR/Stat3-Mediated c-Myc Downregulation

**DOI:** 10.3390/ijms222312961

**Published:** 2021-11-30

**Authors:** Ji-Hyang Kim, Hack-Sun Choi, Dong-Sun Lee

**Affiliations:** 1 Interdisciplinary Graduate Program in Advanced Convergence Technology & Science, Jeju National University, Jeju 63243, Korea; seogwi12@jejunu.ac.kr; 2 Practical Translational Research Center, Jeju National University, Jeju 63243, Korea; 3 Subtropical/Tropical Organism Gene Bank, Jeju National University, Jeju 63243, Korea; choix074@jejunu.ac.kr; 4 Faculty of Biotechnology, College of Applied Life Sciences, Jeju National University, SARI, Jeju 63243, Korea; 5 Bio-Health Materials Core-Facility Center, Jeju National University, Jeju 63243, Korea

**Keywords:** triple-negative breast cancer, primaquine, nuclear EGFR, Stat3, early endosome, c-Myc

## Abstract

Triple-negative breast cancer (TNBC) cells overexpress the epidermal growth factor receptor (EGFR). Nuclear EGFR (nEGFR) drives resistance to anti-EGFR therapy and is correlated with poor survival in breast cancer. Inhibition of EGFR nuclear translocation may be a reasonable approach for the treatment of TNBC. The anti-malarial drugs chloroquine and primaquine have been shown to promote an anticancer effect. The aim of the present study was to investigate the effect and mechanism of chloroquine- and primaquine-induced apoptosis of breast cancer cells. We showed that primaquine, a malaria drug, inhibits the growth, migration, and colony formation of breast cancer cells in vitro, and inhibits tumor growth in vivo. Primaquine induces damage to early endosomes and inhibits the nuclear translocation of EGFR. Primaquine inhibits the interaction of Stat3 and nEGFR and reduces the transcript and protein levels of c-Myc. Moreover, primaquine and chloroquine induce the apoptosis of breast cancer cells through c-Myc/Bcl-2 downregulation, induce early endosome damage and reduce nEGFR levels, and induce apoptosis in breast cancer through nEGFR/Stat3-dependent c-Myc downregulation. Our study of primaquine and chloroquine provides a rationale for targeting EGFR signaling components in the treatment of breast cancer.

## 1. Introduction

Triple-negative breast cancer (TNBC) lacks estrogen receptor, progesterone receptor, and human epidermal growth factor receptor 2 (HER2) expression and accounts for 15% to 20% of breast cancers [1]. TNBC patients have a higher rate of relapse and a worse prognosis than other breast cancer patients [2]. Several research groups have shown that the molecular therapeutic target of TNBC is an epidermal growth factor receptor (EGFR) and that EGFR is overexpressed in TNBC [3,4]. EGFR has two functions: membrane-bound EGFR and nuclear EGFR (nEGFR) [5]. The classic EGFR protein is a cell-membrane-bound receptor tyrosine kinase (RTK) that initiates growth and survival signal pathways [6,7]. Twenty years ago, nEGFR was observed in hepatocytes and was found to be involved in transcriptional regulation, cell proliferation, DNA replication, and chemo- and radioresistance [8,9]. To understand EGFR biology, it is important to study the intracellular trafficking pathway mediating the nuclear translocation of EGFR [8,10]. EGF stimulation of plasma-membrane-located EGFR induces the dimerization of EGFR and its internalization into endosomes. Then, EGFR can be translocated into the Golgi/endoplasmic reticulum (ER). EGFR moves from the endoplasmic reticulum (ER) into the nucleus [11,12,13,14]. nEGFR, as a molecular target in cancer, functions as a cotranscription factor for the expression of several oncogenes: cyclin D1, inducible nitric oxide synthase (iNOS), B-Myb, Aurora kinase A, cyclooxygenase 2 (COX2), c-Myc, breast cancer resistance protein (BCRP), and signal transducer and activator of transcription 1 (Stat1) [15,16,17,18,19,20,21]. nEGFR activates DNA-dependent protein kinase (DNA-PK) to enhance DNA repair [22]. These data suggest that tumors are dependent on two distinct compartments of EGFR signaling to give oncogenic properties: membrane-bound EGFR signaling and nEGFR signaling [5]. Cancer cells that are resistant to cetuximab, a monoclonal antibody that binds to EGFR, express high levels of nEGFR and Src family kinase (SFK) [23,24]. The SFK-dependent phosphorylation of EGFR, at tyrosine 1101, plays an important role in the initiation of EGFR nuclear translocation [25]. EGFR is a therapeutic target in cancer patients. However, previous studies have shown that TNBC is resistant to the EGFR-targeting antibody cetuximab or EGFR inhibitors (tyrosine kinase inhibitors, TKIs). The nuclear translocation of EGFR reduces the inhibitory effects of cetuximab and confers AXL RTK-mediated cetuximab resistance through the activation of downstream signaling [5,10].

The internalization of ligand-induced EGFR is mediated by clathrin- and lipid-raft-mediated endocytosis [26]. Inhibition of EGFR endocytosis decreased the proliferation and induced the apoptosis of cancer cells. Rab25 plays a critical role in EGFR endocytosis. EGFR endocytosis is a novel therapeutic target in cancer with wild-type EGFR [27].

In this study, primaquine, a malaria drug, was found to induce early endosome damage and reduce nEGFR; primaquine was also found to induce apoptosis levels in breast cancer through nEGFR/Stat3-dependent c-Myc downregulation. Our data suggest that primaquine can be used in breast cancer treatment as it targets the nuclear translocation of EGFR.

## 2. Results

### 2.1. The Antimalarial Drug Primaquine Decreases Breast Cancer Cell Viability and Inhibits Tumor Growth in a Mouse Xenograft Model

**Figure 1:**
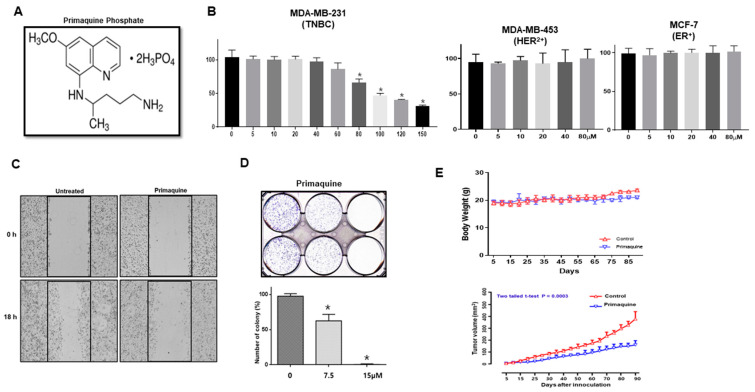
Effect of primaquine on breast cancer hallmarks and on tumor growth in a nude mouse model. (**A**) Molecular structure of primaquine phosphate. (**B**) The proliferation of breast cancer cells was measured using an MTS assay kit and the CellTiter 96 Aqueous One Solution kit. Breast cancer cells were incubated in 96-well plates in the presence of primaquine (5, 10, 20, 40, 80, 100, 120, and 150 μM and DMSO). Values are the mean ± standard deviation (SD) of 3 independent experiments. * indicates *p* < 0.05 vs. control. (**C**) Effect of primaquine on the migratory ability of breast cancer. The migration of MDA-MB-231 cancer cells with/without primaquine was photographed at 0 and 18 h. (**D**) Effect of primaquine on the colony formation of breast cancer cells. Two thousand MDA-MB-231 cells were cultured in 6-well plates with/without primaquine at the indicated concentrations for one week. Representative image of colonies. Values are mean ± SD of 3 independent experiments. * indicates *p* < 0.05 vs. control. (**E**) The effect of primaquine on tumor growth in a xenograft mouse model. A total of 2 × 10^6^ cancer cells were injected into the mammary fat pad of each nonobese diabetic/severe combined immunodeficiency (NOD/SCID) female nude mouse. Effect of tumor growth on primaquine and MDA-MB-231 cell-bearing immunodeficient nude mice. The dose of drug used was 2 mg/kg once every 10 days. Tumor volume was measured once every 10 days using a caliper and calculated as (width^2^ × length)/2. Tumor growth curves were monitored during the experimental period.

To examine the effect of primaquine on the viability of TNBC cells, MDA-MB-231 cells and HCC1937 cells were investigated by a 3-(4,5-Dimethylthiazol-2-yl)-5-(3-carboxymethoxyphenyl)-2-(4-sulfophenyl)-2H-tetrazolium (MTS) assay, which detects cell proliferation. Compared to those with dimethyl sulfoxide (DMSO), the live breast cancer cells incubated with primaquine were significantly decreased (IC_50_: 81.2 µM) (Figure 1A,B and Supplementary Figure S1). However, primaquine showed no inhibitory effect on the proliferation of MCF-7 (ER+) and MDA-MB-453 (HER2+) cells (Figure 1B). Next, we assayed the effects of primaquine on cell migration and colony formation. Primaquine reduced the cell migration and colony formation of breast cancer cells (Figure 1C,D). These results suggest that primaquine directly controls cell growth and biological behaviors in breast cancer. Based on the inhibitory effects of primaquine on cell proliferation, we examined the effect of primaquine on tumor growth in a xenograft model of breast cancer. As shown in Figure 1E, tumor-bearing mice were treated with or without primaquine (2 mg/kg) once every 10 days for 90 days. Primaquine treatment inhibited tumor growth, as indicated by a decrease in the volume of the observed tumors (Figure 1E). Mice in the primaquine-treated group and control group showed similar body weights (Figure 1E). Our data indicates that primaquine inhibits tumor growth in a xenograft mouse model.

### 2.2. Primaquine Affects the Endolysosomal System and Impairs the Endocytosis-Mediated Degradation of EGFR

**Figure 2:**
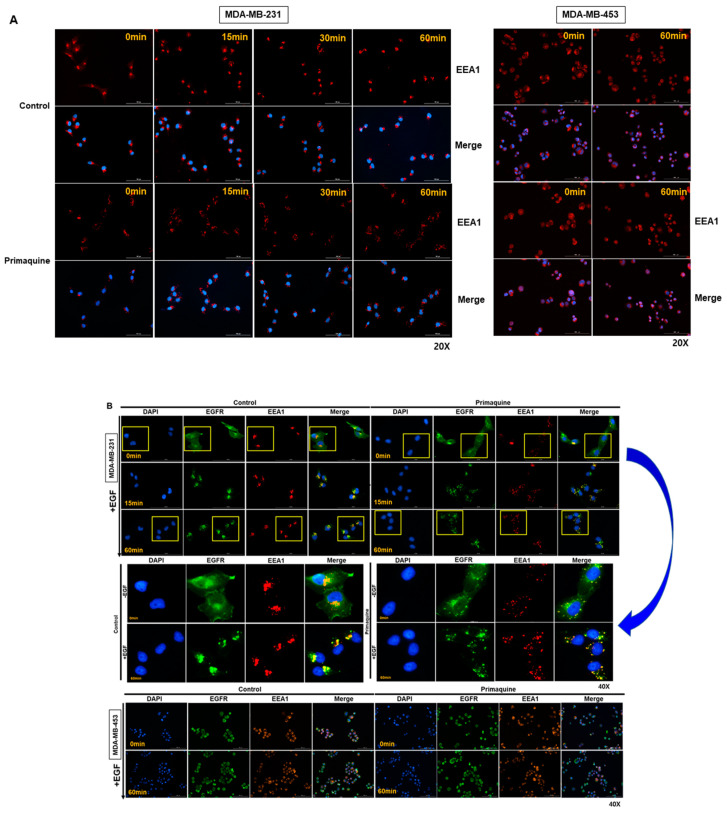
Primaquine has an impact on the endosome system and impairs the endocytosis-mediated degradation of EGFR. (**A**) MDA-MB-231 and MDA-MB-453 cells were exposed to DMSO or 50 μM primaquine for 1 h before processing for immunofluorescence microscopy and stained with anti-EEA1 and DAPI. (**B**) MDA-MB-231 and MDA-MB-453 cells were exposed to 50 µM primaquine or DMSO for 1 day and then treated with EGF from 0 to 60 min. Before processing for immunofluorescence microscopy, cancer cells were stained with anti-EEA1 and anti-EGFR. Insets represent high magnification images of the region indicated by the yellow rectangle.

As a malaria drug, chloroquine (CQ) can affect the function of the endolysosomal system and impair the endocytosis-mediated degradation of EGFR [28]. We assessed the early endosome protein marker EEA1 upon treatment with primaquine by immunofluorescence microscopy. When MDA-MB-231(TNBC) and MDA-MB-453 (HER2+) cells were exposed to primaquine (Figure 2A,B), the distribution of EEA1 on MDA-MB-231 cells was changed, and its signal became faint over time. However, the distribution of EEA1 (early endosome marker) on MDA-MB-453 cells was not changed. Our results showed that primaquine alters the endolysosomal system of TNBC cells. In the control cells, EGFR was rapidly internalized following EGF treatment, and primaquine treatment decreased the EGFR internalization rate after 15 min of EGF treatment (Figure 2B). Primaquine did not changed EGFR internalization of EGF treatment on MDA-MB-453 cells (HER2+), but it changed EGFR internalization of EGF treatment on MDA-MB-231 cells (TNBC). Our immunofluorescence staining of EGFR showed its perinuclear accumulation in Figure 2B. This data showed disrupted endocytic trafficking. We showed that primaquine dysregulated the endolysosomal system and EGFR endosomal trafficking, and induced the endocytosis-mediated degradation of EGFR in TNBC cells.

### 2.3. Primaquine Reduces the Expression of nEGFR in Breast Cancer

**Figure 3:**
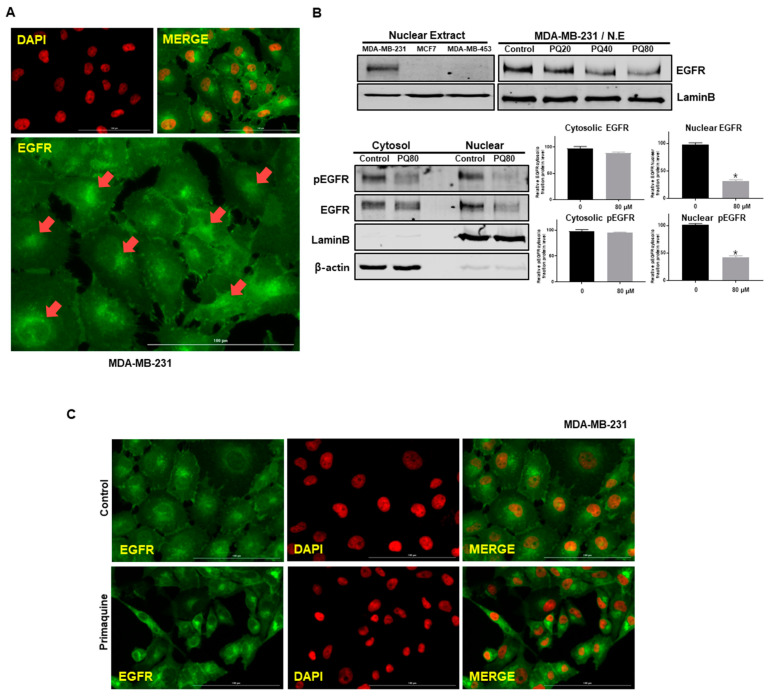
nEGFR expression was reduced in MDA-MB-231 cells upon primaquine treatment. (**A**) Nuclear localization of EGFR with anti-EGFR (green) and nuclei (red, DAPI) using a fluorescence microscope (Lionheart FX, BioTek, Winooski, VT, USA). nEGFR resulted from merging of EGFR (green) and DAPI (red). (**B**) MCF-7, MDA-MB-453, and MDA-MB-231 cells were treated with primaquine (20, 40, and 80 μM) for 24 h and subjected to Western blot analysis. Cells were finally lysed, and cytosolic and nuclear proteins were isolated. The cytosolic and nuclear proteins were identified with anti-pEGFR and anti-EGFR antibodies by Western blotting. The expression of lamin B and β-actin were determined as a loading control for nuclear and cytosolic lysates. Values are the mean ± SD of 3 independent experiments. * indicates *p* < 0.05 vs. control. (**C**) Cancer cells with/without primaquine were washed, fixed, permeabilized, and blocked with 0.1% normal goat serum for 60 min. Cells were incubated with primary monoclonal EGFR antibody. Immunostained cells were examined with a fluorescence microscope (Lionheart FX, BioTek, Winooski, VT, USA). Red, nuclei stained by DAPI; green, EGFR. nEGFR was identified by merging EGFR (green) and DAPI (red) images.

Breast cancer cells overexpress EGFR, and EGFR has two functions: membrane-bound signaling and nuclear signaling. nEGFR enhances resistance to anti-EGFR therapies and is a functional molecular target in TNBC [5]. As primaquine impairs the endocytosis-mediated degradation of EGFR, we assessed nEGFR expression in breast cancer. MCF-7 (ER+) and MDA-MB-453 (HER2+) cells did not show nEGFR expression according to Western blotting (Figure 3B). MDA-MB-231 cells exhibited nEGFR expression according to immunofluorescence microscopy and Western blotting (Figure 3A,B). After treatment with primaquine, we again examined nEGFR expression in breast cancer cells. The levels of nEGFR were decreased in a primaquine-concentration-dependent manner (Figure 3B), and lower levels of nEGFR were also confirmed by immunofluorescence microscopy (Figure 3C). We examined the localization of EGFR after treatment with primaquine on MDA-MB-231 and HCC-1937 cells; primaquine reduced the cytosolic and nuclear fraction of EGFR and pEGFR (Figure 3B and Supplementary Figure S1C).

### 2.4. Primaquine Regulates EGFR Phosphorylation and the EGFR Downstream Signaling Pathway

**Figure 4:**
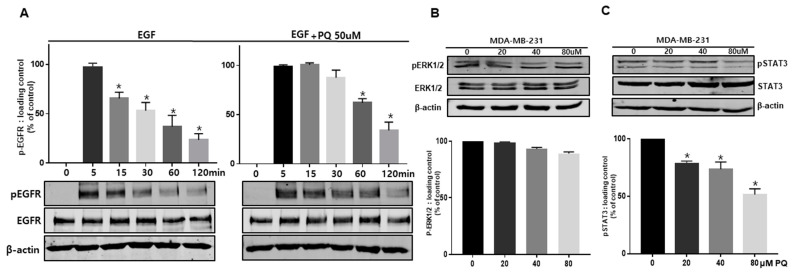
Primaquine treatment delayed EGFR signaling and reduced Stat3 signaling. (**A**) MDA-MB-231 cells were exposed to DMSO or 50 µM primaquine for 24 h and then treated with EGF from 0 to 120 min. Cells were finally lysed and proteins were identified with anti-pEGFR and anti-EGFR antibodies by Western blotting. (**B**) MDA-MB-231 cells were exposed to DMSO or 20, 40, and 80 µM primaquine for 24 h. Cancer cells were finally lysed and proteins were identified with anti-pERK1/2 and anti-ERK1/2 antibodies by Western blotting. (**C**) MDA-MB-231 cells were exposed to DMSO or 20, 40, and 80 µM primaquine for 24 h. Cancer cells were finally lysed and proteins were identified with anti-pStat3 and anti-Stat3 antibodies by Western blotting. The data are presented as the mean ± SD; *n* = 3; * indicates *p* < 0.05 vs. control.

To assess the effect of primaquine on EGFR phosphorylation and the EGFR downstream signaling pathway, we analyzed the activity of EGFR and its downstream signaling proteins, such as ERK and Stat3. Primaquine treatment combined with EGF exhibited a prolonged increase in EGFR phosphorylation compared to EGF treatment alone (Figure 4A). Although the autophosphorylation of EGFR disappeared over time in the control, the turnover of pEGFR was delayed in primaquine-treated cells (Figure 4A). To confirm EGFR downstream signaling pathways, breast cancer cells were treated with primaquine. Primaquine treatment was involved in regulating Stat3 signaling but not ERK signaling (Figure 4B,C). These data reveal that the combination of EGF and primaquine treatment in breast cancer resulted in a prolonged increase in EGFR phosphorylation. Primaquine might dysregulate EGFR signaling and downregulate the Stat3 signaling pathway.

### 2.5. The nEGFR Protein Physically Interacts with Stat3 and this Interaction Is Inhibited by Primaquine Treatment

**Figure 5:**
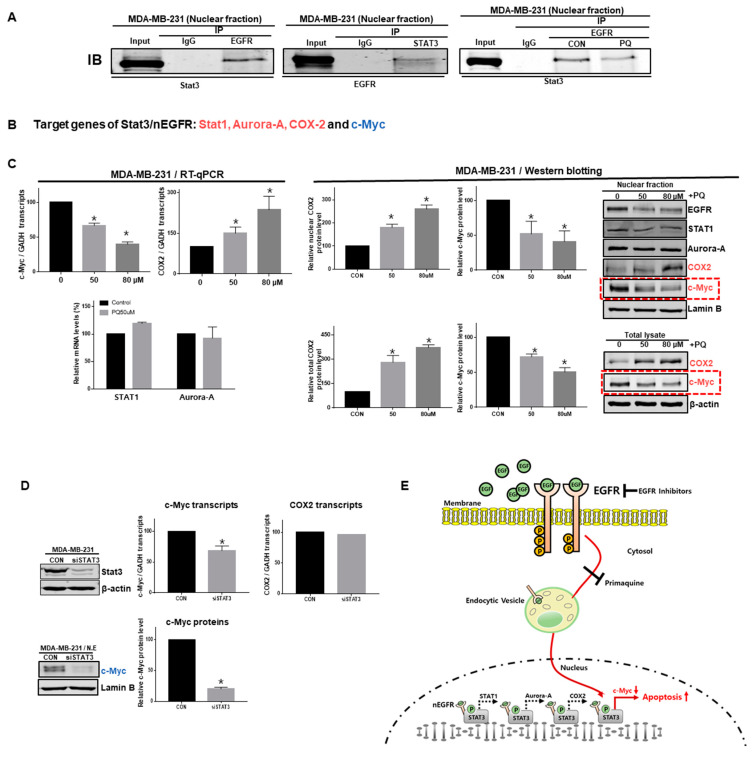
Primaquine reduced the interaction of nEGFR and Stat3 and reduced the transcript and protein levels of c-Myc. (**A**) nEGFR and Stat3 were immunoprecipitated from the nuclear protein extracts of breast cancer cells under primaquine treatment with anti-EGFR or anti-Stat3 antibodies. Western blotting was performed using the indicated antibodies. (**B**) The target genes of the Stat3/nEGFR complex were Stat1, Aurora-A, COX2, and c-Myc. (**C**) Effect of primaquine on transcript levels, total levels, and nuclear levels of the Stat1, Aurora-A, COX2, and c-Myc proteins. Total and nuclear c-Myc protein levels under primaquine (50 and 80 µM) or DMSO treatment conditions. The data are presented as the mean ± SD; *n* = 3; * indicates *p* < 0.05 vs. control. (**D**) siRNA-induced silencing of Stat3 reduced the transcript and protein levels of c-Myc. Representative images of Western blots are shown. Data from triplicate experiments are shown as the mean ± SD. Compared with control, * indicates *p* < 0.05. (**E**) Inhibition of nEGFR function by primaquine. EGF stimulation induces EGFR dimerization and internalization to endocytic vesicles. EGFR then undergoes nuclear translocation. EGFR is consistently detected in the nuclei of breast cancer cells. nEGFR binds to Stat3, and the EGFR/Stat3 complex regulates the transcription of Stat1, Aurora kinase A, COX2, and c-Myc. Primaquine reduces nEGFR expression by inhibiting EGFR trafficking.

As EGFR signaling regulates Stat3 phosphorylation, we assessed the interaction of nEGFR and nuclear Stat3. Immunoprecipitation using anti-EGFR and anti-Stat3 antibodies showed that nEGFR interacts with nuclear Stat3 (Figure 5A). Primaquine inhibited the interaction of Stat3 and EGFR. The nEGFR/Stat3 complex is known to regulate the transcription of the Stat1, Aurora A, COX2, and c-Myc genes (Figure 5B,E) [29]. We assessed the mRNA levels of these four genes under primaquine treatment using RT-qPCR, and only the transcript levels of c-Myc were decreased (Figure 5C). We confirmed that the total and nuclear c-Myc protein levels were decreased by primaquine treatment through Western blot analysis using an anti-c-Myc antibody (Figure 5C). To confirm that nuclear Stat3 regulates c-Myc, we downregulated the Stat3 gene using si-Stat3. Stat3 downregulation induced a decrease in the c-Myc gene (Figure 5D). Our data showed that primaquine decreases c-Myc gene expression by inhibiting the formation of the Stat3/nEGFR complex (Figure 5E).

### 2.6. Primaquine Induces the Apoptosis of Breast Cancer Cells through nEGFR/Stat3-Mediated c-Myc and Bcl-2 Downregulation

**Figure 6:**
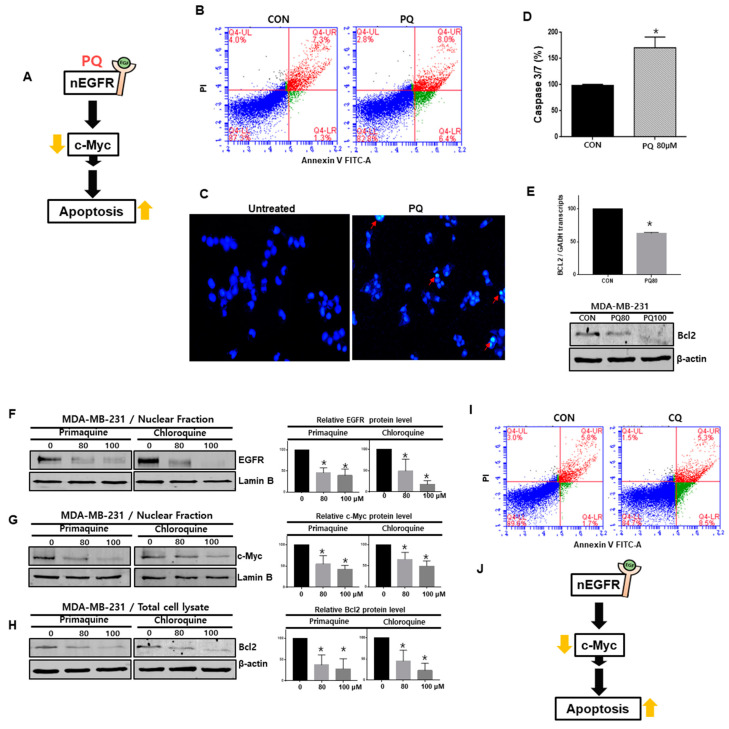
Primaquine and CQ induced the apoptosis of breast cancer through nEGFR and c-Myc regulation. (**A**) The proposed model for breast cancer cell death induced by primaquine. (**B**) Primaquine (80 μM) induced the apoptosis of cancer cells. Cells undergoing primaquine-induced apoptosis were analyzed by using an annexin V-PI staining kit. (**C**) Analysis of apoptotic cancer cells by fluorescence staining. The nuclei of breast cancer cells were stained with Hoechst 33258 (magnification, ×100). The red arrows indicate apoptotic bodies. (**D**) The caspase-3/7 activity of breast cancer cells was determined with the Caspase-Gloss 3/7 kit. (**E**) Effect of primaquine (0, 80, and 100 μM) on Bcl-2 protein levels in breast cancer cells. (**F**–**H**) Effect of primaquine and CQ (0, 80, and 100 µM) on nEGFR, cMyc and Bcl-2 protein levels in breast cancer cells. (**I**) CQ (80 μM) induced the apoptosis of cancer cells. Cells undergoing CQ-induced apoptosis were analyzed by using an annexin V-PI staining kit. (**J**) The proposed model for CQ-induced breast cancer cell death. The data are presented as the mean ± SD; *n* = 3; * indicates *p* < 0.05 vs. control.

To examine the mechanism of breast cancer cell death (MDA-MB-231 and HCC-1937) caused by primaquine treatment, we assessed apoptosis. Primaquine induced the early and late apoptosis of breast cancer cells and induced apoptosome formation (Figure 6B,C and Supplementary Figure S1B). We examined the apoptosis-related enzyme caspase-3/7. Primaquine increased the activity of caspase-3/7 and decreased Bcl-2 levels (Figure 6D,E). Our results showed that primaquine induces the apoptosis of cancer cells by reducing c-Myc and Bcl-2 expression (Figure 6A).

As chloroquine (CQ), together with primaquine, is the first-line treatment recommended for malaria and has been reported to promote anticancer activity and regulate the endocytosis-mediated degradation of EGFR [28,30], we assessed the effects of CQ on breast cancer apoptosis. CQ induced the early and late apoptosis of breast cancer cells (Figure 6I). To understand the apoptosis mechanism caused by CQ, we treated breast cancer cells with CQ. CQ and primaquine reduced the protein levels of nEGFR, nuclear c-Myc, and total Bcl-2 (Figure 6F–H). Our data showed that CQ, similar to primaquine, induces apoptosis through the nEGFR/c-Myc/Bcl-2 pathway (Figure 6J).

## 3. Discussion

The antimalarial drug CQ exerts anti-breast-cancer properties by modulating the microenvironment and inducing apoptosis. CQ may have potential in breast cancer therapy [30]. The antimalarial drug CQ is promising for cancer treatment, and several clinical trials have shown its favorable effects as a novel antitumor drug [31]. Primaquine, sahaquine, and hybrid sahaquine and primaquine dimers also have potential as anticancer agents [32,33]. CQ and hydroxychloroquine have substantial antineoplastic effects in preclinical models and are involved in mechanisms other than autophagy inhibition [34]. Cotreatment with CQ and primaquine sensitized drug-resistant cancer cells through p-glycoprotein inhibition [35]. The antineoplastic mechanism of primaquine was previously unclear, but here we have shown a new mechanism of primaquine: it exerts anticancer effects through nEGFR downregulation (Figure 1 and Figure 3). CQ disrupted the endolysosomal system both in vitro and in vivo and induced the disappearance of the early endosome membrane protein EEA1. EGFR levels were decreased under CQ treatment [28]. As primaquine induced the apoptosis of breast cancer, we wanted to identify the apoptotic mechanism of primaquine. We found that primaquine dysregulated the endolysosomal system and EGFR endosomal trafficking, and induced the endocytosis-mediated degradation of EGFR (Figure 2). Our data showed that breast cancer cells express nEGFR and that nEGFR expression was reduced under primaquine treatment (Figure 3 and Figure 5C).

EGFR regulates epithelial tissue development and homeostasis. However, EGFR induces tumorigenesis in lung cancer, breast cancer, and glioblastoma [36]. EGFR functions as a cytoplasmic-membrane-bound RTK that induces growth and survival signals in cancer [6]. Recently, studies have shown that EGFR can be localized to, and function in, the nucleus [11]. nEGFR drives resistance to cetuximab in TNBC. In a breast cancer study, nEGFR was detected in a TNBC cell line and human tumors [5]. In the breast cancer cohort, 40% of patients showed nEGFR positivity, which was correlated with a worse survival rate [37,38]. Research papers have suggested that nEGFR regulates cyclin D1, iNOS, B-Myb, cyclooxygenase-2 (COX-2), aurora kinase A, c-Myc, BCRP, and Stat1 [29]. A previous study showed that the nuclear interaction of Stat3 and EGFR occurs when the iNOS/NO pathway is activated in breast cancer [16]. We assessed the nuclear interaction of Stat3 and EGFR. We confirmed the nuclear interaction of Stat3 and EGFR, and the interaction was reduced under primaquine treatment. Previously, another group showed that the nEGFR/Stat3 complex regulated iNOS, COX-2, aurora kinase A, c-Myc, and Stat1 [11]. Our data showed that primaquine downregulated the c-Myc gene through the regulation of the nEGFR/Stat3 complex and induced the apoptosis of breast cancer cells (Figure 5 and Figure 6). Exposure of Barrett’s and esophageal adenocarcinoma cells to acidic bile salts activated EGFR-Stat3 signaling (EGFR-STAT3 protein complex) and induced the expression of Stat3 target genes (IL-6, IL-17A, BCL-xL, Survivin, and c-MYC) [39]. Our data showed that Stat3 downregulation using siRNA decreased the expression of the c-Myc gene but not the COX2 gene. Stat3/nEGFR regulated the c-Myc gene and induced apoptosis (Figure 6). Primaquine reduced the interaction of Stat3/nEGFR and reduced the levels of c-Myc transcripts and proteins. Finally, primaquine induced apoptosis through c-Myc downregulation. c-Myc has a main function in growth regulation, differentiation, and apoptosis [40]. c-Myc regulates important biological pathways involved in the growth and proliferation of tumor cells [41]. c-Myc inhibitors are important therapeutics for cancer. We found that primaquine and CQ, which are malaria drugs, induce apoptosis through the nEGFR/Stat3 complex and c-Myc downregulation. Taken together, these results indicate that primaquine induces damage to early endosomes and inhibits the nuclear translocation of EGFR. Primaquine inhibits the interaction of Stat3/nEGFR and reduces the transcript and protein levels of c-Myc. Primaquine and CQ induce the apoptosis of breast cancer through nEGFR/Stat3-dependent c-Myc downregulation, which provides a strategy for the treatment of breast cancer by targeting the EGFR signaling components.

## 4. Materials and Methods

### 4.1. Reagents and Antibodies

Primaquine (160393) and chloroquine (CQ; PHR1258) were purchased from Sigma (St. Louis, MO, USA). Anti-EGFR (#4267), anti-pEGFR (#2234), anti-pSTAT3 (#9145), anti-cMyc (#5605), anti-STAT1 (#9167), anti-Aurora A (#14475), and anti-Bcl-2 (#4223) were obtained from Cell Signaling Technology (Beverly, MA, USA). Anti-STAT3 (SC-482), anti-β-actin (SC-47778), anti-COX2 (SC-19999), and anti-Lamin B (SC-6216) were purchased from Santa Cruz (CA, USA). The antibody for immunofluorescence was anti-EEA1 (610457, BD Transduction Laboratory, Franklin Lakes, NJ, USA).

### 4.2. Cell Lines and Media

Human MDA-MB-231, MCF-7, HCC1937 cells, and MDA-MB-453 breast cancer cells were purchased from the Korean Cell Line Bank (Seoul, Korea). MDA-MB-231 and HCC1937 cells were cultured in Dulbecco’s modified Eagle’s medium (DMEM, Gibco, Thermo Fisher Scientific, Waltham, MA, USA) supplemented with 10% fetal bovine serum (Gibco, Thermo Fisher Scientific), penicillin (100 units/mL), and streptomycin (100 μg/mL) at 37 °C with 5% CO_2_. MDA-MB-231 cells were seeded in a 6-well plate at a density of 2 × 10^5^ cells/well.

### 4.3. Cell Proliferation Assay

We followed a previously reported method [42]. MDA-MB-231, HCC1937 cells, MCF-7, and MDA-MB-453 cells were seeded at a density of 1.5 × 10^6^ cells/plate in a 96-well plate and cultured for 24 h, and we treated cells with primaquine for 24 h. We followed the CellTiter 96^®^ Aqueous One Solution cell kit protocol and used SpectraMax to read the optical density at 490 nm.

### 4.4. Clonogenic and Scratch Assays

The cells were cultured in a 6-well plate at a density of 1 × 10^3^ cells/well for 1 day and treated with different concentrations of primaquine. The cancer cells were incubated for 7 days, and the colonies were counted. The cells were seeded at a density of 1.5 × 10^6^ cells/plate in a 6-well plate, and scratches were made using a 10-μL micropipette tip. After 24 h, the cells were washed with DMEM to remove debris and were then treated with primaquine. We followed a previously described method [43]. The wound areas were photographed under a microscope at 10× after 18 h.

### 4.5. Xenograft Transplantation

Twelve 4-week-old nude mice were injected with MDA-MB-231 cells and treated with primaquine (2 mg/kg). Tumor volumes were measured after 90 days using a formula. Mouse experiments were performed as described previously [44]. Animal care and animal experiments were performed in accordance with protocols approved by the Jeju National University Animal Care and Use Committee (JNU-IACUC; Approval Number 2020-035). Female nude mice (4 weeks old) were purchased from OrientBio (Seoul, Korea) and kept in mouse facilities for 10 days.

### 4.6. Western Blot Analysis

Protein samples isolated from breast cancer were separated using 10% sodium dodecyl sulfate-polyacrylamide gel electrophoresis (SDS-PAGE) and transferred to a PVDF membrane (Millipore, Burlington, MA, USA). Blots were blocked in Odyssey blocking buffer at room temperature for 60 min. The blots were incubated overnight with primary antibodies at 4 °C. After membranes were washed, the blots were incubated with IRDye 680RD and 800W secondary antibodies, and signals were detected using ODYSSEY CLx (Li-Cor, Lincoln, NE, USA).

### 4.7. EGFR Degradation and Trafficking

MDA-MB-231 and MDA-MB-453 cells were cultured in 96-well plates on glass coverslips for EEA1 and EGFR trafficking. MDA-MB-231 cancer cells were treated with 50 µM primaquine for 2 h. After treatment, we incubated them for 0, 15, 30, or 60 min with 75 ng/mL EGF. Finally, we analyzed the samples by immunofluorescence.

### 4.8. Immunofluorescence

We followed a previously reported method [45]. MDA-MB-231and MDA-MB-453 cancer cells were fixed using 4% paraformaldehyde for 20 min, permeabilized with 0.5% Triton X-100 for 15 min, blocked with 3% bovine serum albumin (BSA) for 1 h, stained with primary antibody, and then conjugated to secondary anti-mouse Alexa555 (A32727, Thermo Fisher, Waltham, MA, USA) and rabbit Alexa488 (A32731, Thermo Fisher, Waltham, MA, USA) antibodies. 4′,6-Diamidino-2-phenylindole (DAPI) solution was used to stain the nuclei of cancer cells. Finally, EGFR and EEA1 were visualized using a fluorescence microscope (Lionheart FX live cell imager, BioTek).

### 4.9. Immunoprecipitation

MDA-MB-231 cells were washed with 1× PBS and resuspended in IP lysis buffer. The lysate was incubated with anti-STAT3 and anti-EGFR antibodies for 24 h at 4 °C. Protein A/G agarose (GenDepot, DAWINbio, Hanam, Korea) was added to the mixtures. All mixtures were centrifuged, washed with IP lysis buffer 3 times, run on SDS-PAGE gels, and subjected to Western blotting.

### 4.10. Gene Expression Analysis

We isolated total RNA from MDA-MB-231 cells. We carried out real-time quantitative RT-PCR using the One Step SYBR PrimeScript RT-PCR kit with SYBR green (Takara, Tokyo, Japan). The primers were purchased from Bioneer Corp. (Daejeon, Korea). PCR was conducted in 10 μL of 2X One Step SYBR RT-qPCR Buffer, 1 μL PrimeScript One Step Enzyme Mix, 1 µg of RNA, and primers for a final volume of 20 µL per reaction. We used a previously described method [42].

### 4.11. Small Interfering RNA (siRNA)

To examine the function of c-Myc and STAT3, we treated MDA-MB-231 cells with human c-Myc and STAT3 siRNA. STAT3 siRNA (NM_003150.3, NM_139276.2, NM_213662.1) and c-Myc siRNA (NM_001354870.1, NM_002467.5) were obtained from Bioneer Corp. (Daejeon, Korea). For siRNA transfection, MDA-MB-231 cells were cultured in 6-well plates for 1 day and transfected using Lipofectamine 3000 (Invitrogen, Carlsbad, CA, USA). The level of protein was investigated by Western blotting.

### 4.12. Hoechst 33342 Staining and Annexin V/PI Assay

MDA-MB-231 cells were treated with 20 µM primaquine and 50 µM CQ for 24 h. After drug treatment, the cells were stained with Hoechst 33342 solution for 30 min at 37 °C and then observed using a microscope (Lionheart FX live cell imager, BioTek). Apoptosis assays were performed using an annexin V/propidium iodide (PI) staining kit (BD, San Jose, CA, USA). The stained samples were analyzed with an Accuri C6 cytometer (BD, San Jose, CA, USA).

### 4.13. Statistical Analysis

Statistical analysis was performed with GraphPad Prism 7 software (GraphPad Prism Inc., San Diego, CA, USA). All data are shown as the mean ± standard deviation. Data from three independent experiments were analyzed using a one-way ANOVA. Differences with a p-value less than 0.05 were considered significant.

## Data Availability

The data presented in this study are available on request from the corresponding author.

## Supplementary Materials

The following are available online at https://www.mdpi.com/article/10.3390/ijms222312961/s1.
