# Supplementary material for: Primaquine Inhibits the Endosomal Trafficking and Nuclear Localization of EGFR and Induces the Apoptosis of Breast Cancer Cells by Nuclear EGFR/Stat3-Mediated c-Myc Downregulation
Source: Int J Mol Sci. 2021 Nov 30;22(23):12961. doi: 10.3390/ijms222312961 (PMC8657416; doi:10.3390/ijms222312961)
Supplement: Supplementary file 1 [file ijms-22-12961-s001.zip › ijms-1477137-supplementary.pdf]

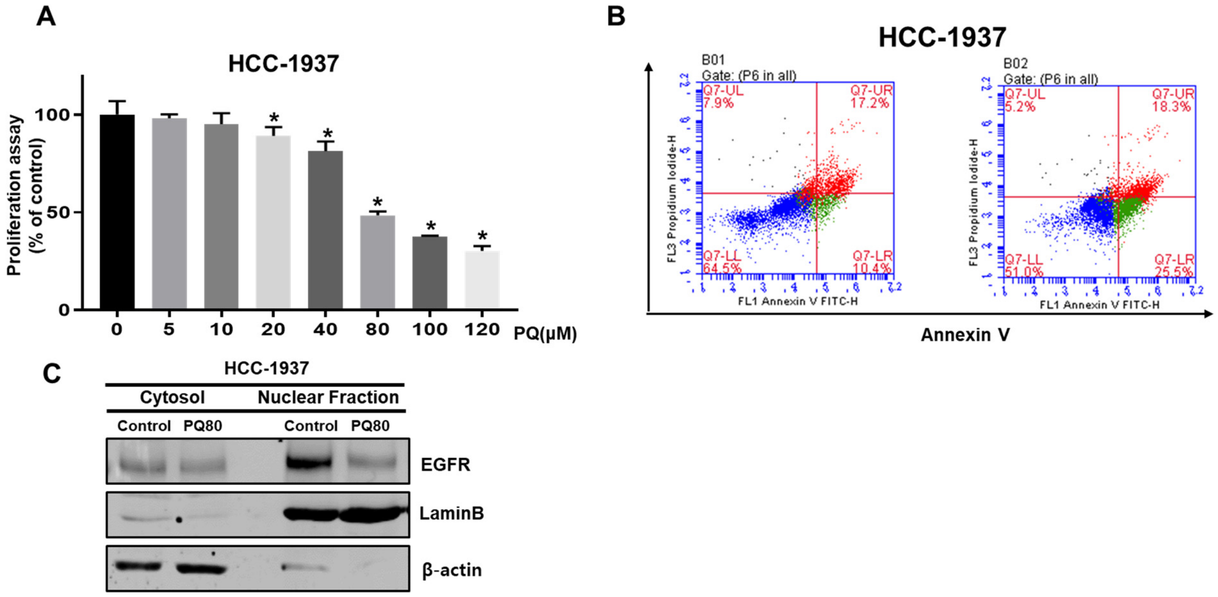


**Supplementary Figure S1.** Effect of primaquine on breast cancer, TNBC HCC-1937. **(A)** The proliferation of breast cancer cells, HCC-1937 was measured using an MTS assay kit, CellTiter 96 Aqueous One Solution kit. Breast cancer cells were incubated in 96-well plates in the presence of primaquine (0, 5, 10, 20, 40, 80, 100, and 120 μM and DMSO). Values are the mean ± SD of 3 independent experiments. *$p < 0.05$ vs. control. **(B)** PQ (control and 80 μM) induced the apoptosis of cancer cells (HCC-1937). Cells undergoing PQ-induced apoptosis were analyzed by using an annexin V-PI staining kit. **(C)** Western blot of EGFR in HCC-1937 under PQ treatment (control or 80 μM).Cytosolic and nuclear EGFR were analyzed by Western blot. Internal control of cytosol and nuclear fraction were used as lamin B and β-actin.
